# Fine tuning the glycolytic flux ratio of EP-bifido pathway for mevalonate production by enhancing glucose-6-phosphate dehydrogenase (Zwf) and CRISPRi suppressing 6-phosphofructose kinase (PfkA) in *Escherichia coli*

**DOI:** 10.1186/s12934-021-01526-1

**Published:** 2021-02-02

**Authors:** Ying Li, He Xian, Ya Xu, Yuan Zhu, Zhijie Sun, Qian Wang, Qingsheng Qi

**Affiliations:** 1 National Glycoengineering Research Center, State Key Laboratory of Microbial Technology, Shandong University, 72 Binhai Dadao, Qingdao, 266237 People’s Republic of China; 2 Marine Biology Institute, Shantou University, Shantou, 515063 People’s Republic of China; 3 School of Chemistry and Molecular Biosciences, Faculty of Science, The University of Queensland, St Lucia, Australia

## Abstract

**Background:**

Natural glycolysis encounters the decarboxylation of glucose partial oxidation product pyruvate into acetyl-CoA, where one-third of the carbon is lost at CO_2_. We previously constructed a carbon saving pathway, EP-bifido pathway by combining Embden-Meyerhof-Parnas Pathway, Pentose Phosphate Pathway and “bifid shunt”, to generate high yield acetyl-CoA from glucose. However, the carbon conversion rate and reducing power of this pathway was not optimal, the flux ratio of EMP pathway and pentose phosphate pathway (PPP) needs to be precisely and dynamically adjusted to improve the production of mevalonate (MVA).

**Result:**

Here, we finely tuned the glycolytic flux ratio in two ways. First, we enhanced PPP flux for NADPH supply by replacing the promoter of *zwf* on the genome with a set of different strength promoters. Compared with the previous EP-bifido strains, the *zwf*-modified strains showed obvious differences in NADPH, NADH, and ATP synthesis levels. Among them, strain BP10BF accumulated 11.2 g/L of MVA after 72 h of fermentation and the molar conversion rate from glucose reached 62.2%. Second, *pfkA* was finely down-regulated by the clustered regularly interspaced short palindromic repeats interference (CRISPRi) system. The MVA yield of the regulated strain BiB1F was 8.53 g/L, and the conversion rate from glucose reached 68.7%.

**Conclusion:**

This is the highest MVA conversion rate reported in shaken flask fermentation. The CRISPRi and promoter fine-tuning provided an effective strategy for metabolic flux redistribution in many metabolic pathways and promotes the chemicals production.

## Background

It is an important challenge in metabolic engineering to reasonably allocate metabolic flux to achieve high yields of target products [1]. Traditional metabolic engineering methods modify and optimize an organism for production of chemicals by decreasing flow through competing pathways and introducing heterogeneous production pathways. As such, metabolic rewiring designs are necessary to increase flux towards essential metabolites, for example, overexpressing native pathways [2, 3], inhibition of competing pathways [4], increasing Coenzyme A (CoA) availability [5], and construction of pyruvate dehydrogenase bypass. Many strategies have been applied to improve production of chemicals [6].

A new strategy is to decrease the generation of harmful byproducts such as CO_2_ or increase the reuse of byproducts by constructing artificial synthetic pathways. With the rapid development of synthetic biology and molecular biotechnology, scientists have made great efforts to maximize microbial chemical yields focusing on enhancing the efficiency of CO_2_ fixation and decreasing CO_2_ emission. Many unnatural pathways have been constructed, such as CETCH [7], MCG [8], NOG [9], MOG [10], and so on. These pathways provide a variety of new ideas to use CO_2_ or one-carbon chemicals as carbon sources, and rewire metabolic pathways [11, 12].

In natural glycolysis, a variety of carbon sources are metabolized through the Embden-Meyerhoff-Parnas (EMP) pathway, which synthesizes C3 (pyruvate) and C2 (acetyl-CoA) metabolites. Acetyl-CoA is a precursor of almost all biosynthesis and energy metabolism pathways. It is normally produced via pyruvate decarboxylation, in which one-third of the carbon is lost as CO_2_. To improve carbon conversion efficiency, Xu et al. constructed the “EP-bifido pathway” in *E. coli* by introducing *fxpk* (encoding bifunctional phosphoketolase) and *fbp* (encoding fructose-1,6-bisphosphatase) to break down fructose 6-phosphate (F6P) into the theoretical maximum amount of acetyl-CoA from glucose. The *edd* gene in the Entner-Doudoroff (ED) pathway and the key enzyme-encoding gene *pfkA* (phosphofructose kinase A) in the EMP pathway were knocked out to save more F6P to break down into C2 metabolites; attenuate the flow of pyruvate to acetyl-CoA; and shift carbon flux from the EMP pathway to the pentose phosphate pathway (PPP) which supplies more NADPH. The EP-bifido pathway achieved high carbon yield of mevalonate (MVA) with 64.3 mol% [13], which is the highest MVA yield that have been reported.

PPP is an important energy metabolism pathway in all organisms. Increase of the dehydrogenase reactions of the PPP is effective in increasing the yield of NADPH-dependent products [14–18]. ^13^C metabolic flux analysis revealed that a MVA producing strain with EP-bifido pathway provided insufficient NADPH from oxidative PPP. In the EP-bifido pathway, the theoretical optimal carbon split between the EMP pathway and PPP in the EP-bifido route for MVA production is 1:6 [13]; thus the resulting maximum carbon MVA theoretical conversion rate of the EP-bifido pathway is 86% (mol/mol). In our previously constructed EP-bifido pathway, the detected carbon split ratio was 0.43:0.57, in which the PPP split ratio was too low. This carbon flux distribution needs to be further optimized.

In addition, the disruption of *pfkA* in the EP-bifido strain caused severe growth defect of the cells. This significantly limited the application of this synthetic pathway. Many reports have demonstrated this limitation of *pfkA* deficiency [19]. Metabolic engineering is usually static by blocking a competing pathway or introducing heterogeneous pathways permanently and continuously [20–22]. Sometimes this has a detrimental effect, for example in the early growth period carbon sources would ideally be dedicated to building biomass. Implementing flexible regulation would be valuable in engineering projects by rebalancing synthetic pathways to respond to the growth phase or the buildup of precursor metabolites [23–25].

Therefore, the artificial pathway must be optimized to be robust and the reducing power should be balanced. To overcome these challenges, here, we designed and constructed fine-tuned EP-bifido *Escherichia coli* strains and enhanced PPP by replacing promoter of *zwf* on the genome to a series of different strength promoters, and suppressing the expression of *pfkA* using the clustered regularly interspaced short palindromic repeats interference (CRISPRi) system. After CRISPRi was applied, we obtained a titer of 8.53 g/L MVA and a yield of 68.7% (mol/mol). This is the highest MVA yield reported in shaken flask fermentation.

## Results

### Enhancement of PPP flux by increasing the expression of *zwf* in different degrees

**Fig. 1:**
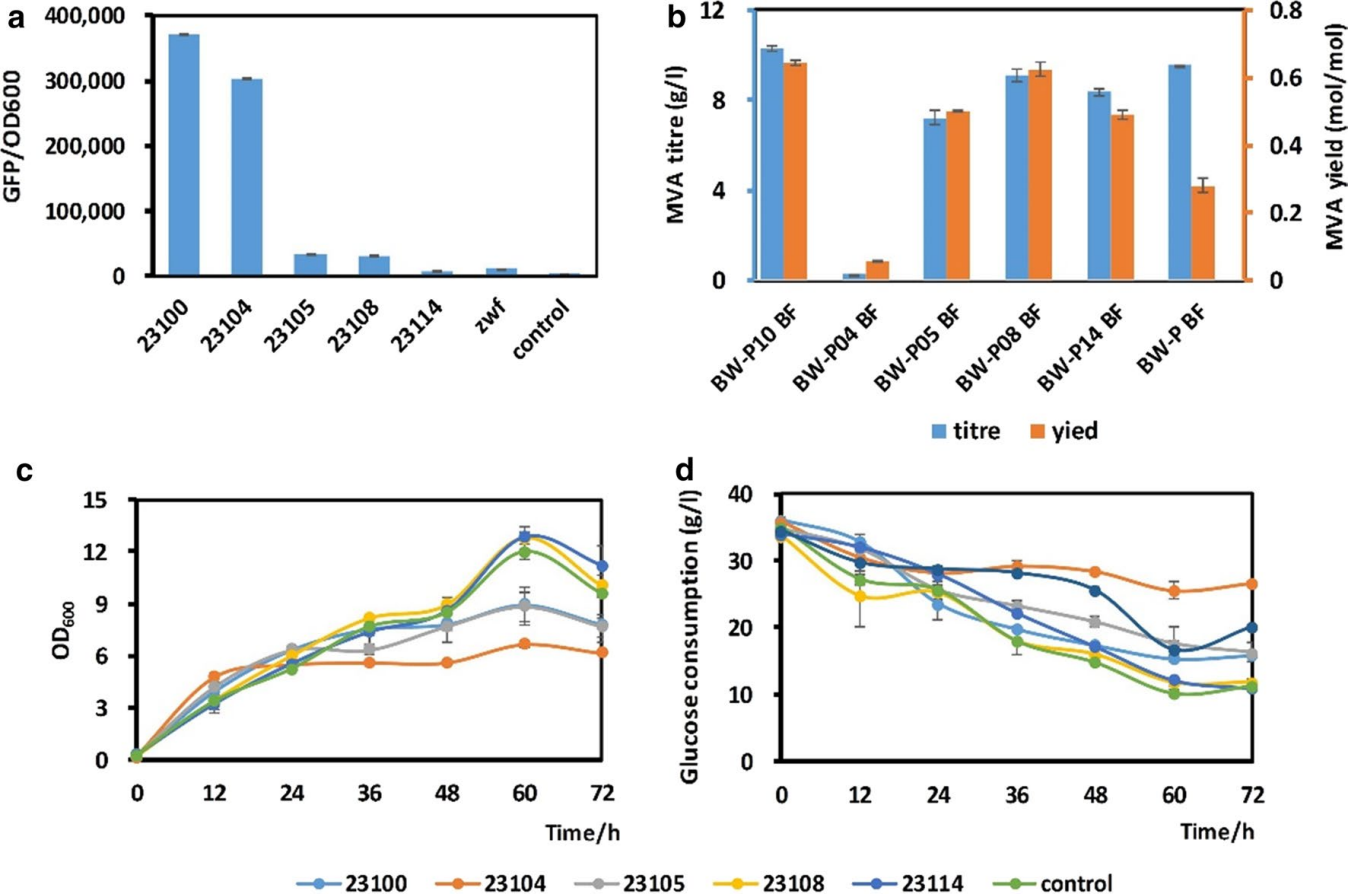
The fermentation results of EP-bifido strains with modified *zwf* promoter replaced by different strength synthetic promoters. **a** The characterization of the strength of a series of synthetic promoters and *zwf* promoter. The fluorescence/OD_600_ of strains was detected at 16 h. MVA titre and molar conversion rate (**b**), cell growth (**c**) and glucose consumption (**d**) during 72 h fermentation

First, to further optimize the flux ratio of EP-bifido pathway, we aimed to enhance the expression of the first key gene of the PPP, *zwf*, by replacing its promoter with relative stronger promoters. We selected five constitutive promoters with different strengths from the Anderson promoter library. The theoretical strength of each promoter is shown in Table 2. We compared the actual expression strength of these five synthetic promoters with the original *zwf* promoter by placing a *gfp* gene downstream of the promoters and cultivated the engineered strains for fluorescence intensity detection. The fluorescence/OD_600_ of detected at 16 h is shown in Fig. 1a. The strength of the promoters was relatively consistent with that stated by the Anderson promoter library. The strength of promoters BBa-J23100 and BBa-J23104 was relatively strong, and BBa-J23100 was the strongest. The strength of the original (native) *zwf* promoter is between that of BBa-J23108 and BBa-J23114, and is relatively weak.

To detect the effect of PPP enhancement on MVA production, plasmids pBSA (expressing three enzymes catalysis acetyl-CoA to mevalonate) and pFF (carrying *fbp* and *fxpk* gene) were transformed into the five *zwf*-enhanced strains and cultivated with the control strain BW-P/pBSA pFF (abbreviated to BW-P BF). Strain BW-P10 BF showed almost the same growth and glucose consumption as the others, while the conversion rate of MVA was far higher than that in the control strain due to less byproducts generation. Promoters BBa-J23100 and BBa-J23108 resulted in the highest yield of MVA, 64.3% (mol/mol) and 62.3% (mol/mol) respectively, although the strength of the promoters did not show a perfectly positive correlation with the MVA yield. This proved that enhancing expression of gene *zwf* was effective for increasing the PPP flux.

### ^13^C-Metabolic flux analysis of changes in central carbon metabolism flux and energy metabolism

**Fig. 2:**
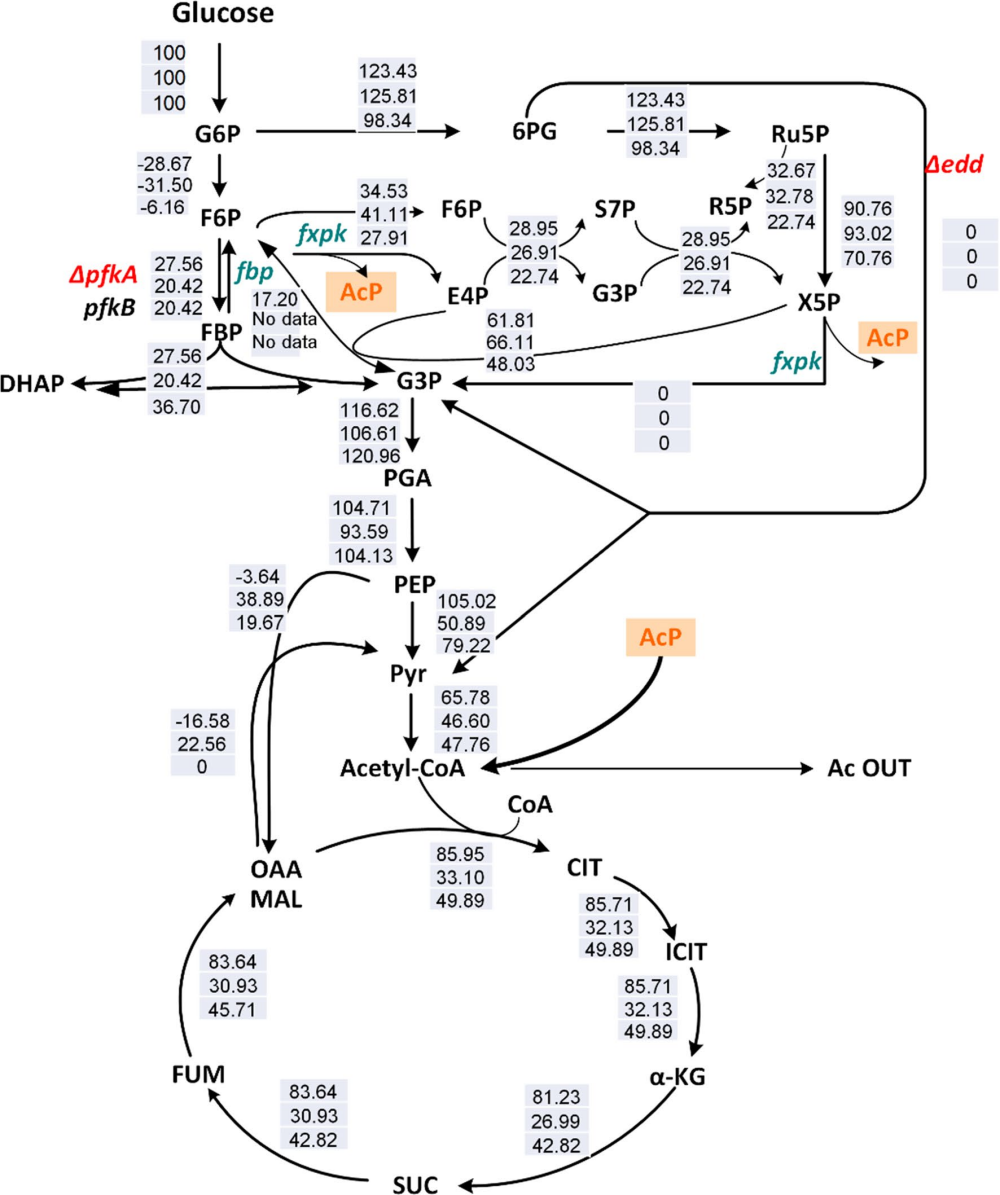
NADH, NADPH and ATP level and its derivations. **a** NADPH generation. **b** NADPH derivations of BW25113, BW-P BF, BW-P08 BF. **c** NADH generation. **d** NADH derivations of BW25113 and BW-P10 BF. **d** ATP generation. **e** ATP derivations. G6PDH, Glucose-6-phoshpate dehydrogenase; ICDH, Isolate dehydrogenase; GAPDH, Glyceraldehyde-3-phosphate dehydrogenase; ME, Malate enzyme; MDH, Malate dehydrogenase; PDH, Pyruate dehydrogenase; KDH, α-Ketoglutarate dehydrogenase; SDH, Succinate dehydrogenase; IDH, Isolate dehydrogenase

Strains BW-P10 BF and BW-P08 BF and control strain BW-P BF were chosen for metabolic flux analysis. With the *zwf* promoter replaced, the normalized data showed that the carbon flux through the oxidative part of the PPP was significantly increased, and the carbon flux through the TCA cycle was decreased, which was consistent with our expectations (Fig. 2). More carbon flux moved towards the EP-bifido pathway (see Additional file 2). The two *zwf*-expression-enhanced strains showed a large difference in TCA cycle flux, which may explain the growth difference between these strains (Fig. 1c).

In addition, the ATP, NADPH, and NADH synthesis capacity and glucose consumption of the three strains were compared based on the ^13^C-MFA data. After the EP-bifido pathway and the MVA synthetic pathway were introduced, the NADPH content and yield of the strain were significantly improved. *pfkA* deficiency shunted carbon flux to the PPP and the expression level of *zwf* was increased. Comparison of the *zwf*-expression-enhanced strains showed that overexpression of *zwf* enhanced NADPH synthesis, and the NADPH level was positively correlated with the promoter strength. Taking strains BW25113, BW-P BF, and BW-P10 BF as examples, the introduction of the EP-bifido pathway and overexpression of *zwf* changed the main source of NADPH: The main NADPH generating pathway shifted from isocitrate dehydrogenase in the TCA cycle to glucose-6-phosphate dehydrogenase in the PPP. This further proved that we have redirected part of the carbon flux of the EMP pathway to the PPP.

In addition, the production of NADH also changed significantly, as shown in Fig. 2c, d. Through *zwf* enhancement, the total amount and the yield of NADH decreased significantly. The NADH was produced distinctly in strain BW-P10 BF compared with wild-type strain BW25113: In strain BW25113, five dehydrogenases were the main source of NADH [glyceraldehyde-3-phosphate dehydrogenase (GAPDH), pyruvate dehydrogenase (PDH), malate dehydrogenase, α-ketoglutarate dehydrogenase, and succinate dehydrogenase]; in strain BW-P10 BF, NADH was mainly formed via GAPDH and PDH.

The EP-bifido pathway and MVA synthetic pathway expression increased the ATP yield from glucose, but the total amount of ATP decreased (Fig. 2e). The *pfkA* deficiency greatly impaired the EMP pathway and thereby blocked the three steps of substrate level phosphorylation absorption (glycerate-1, 3-diphosphate, phosphoenolpyruvate kinase, and succinyl CoA synthetase). This can also be verified from the origin ratio of ATP (Fig. 2f). Enhancing *zwf* expression resulted in increased ATP production and yield in strain BW-P08 BF compared with BW-P BF.

### Down regulation of EMP pathway flux by targeting *pfkA* using CRISPRi system

**Fig. 3:**
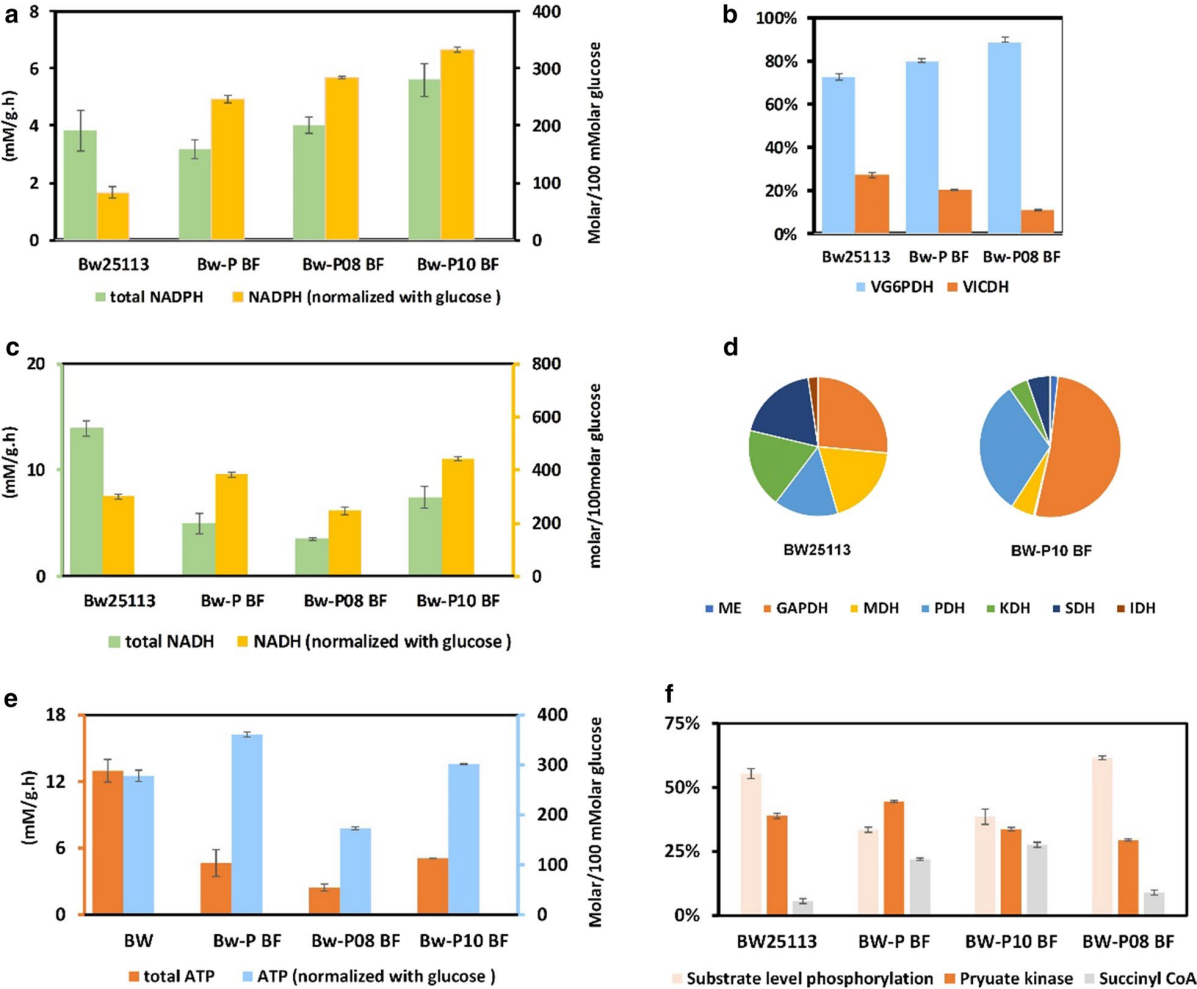
Schematic diagram of CRISPRi on *pfkA* suppression. **a** Schematic diagram of *pfkA* transform. **b** Abridged general view of metabolic flux change after CRISPRi regulation. The red arrow indicated decreased metabolic flux, the green arrow indicated increased metabolic flux

To further rationally use the carbon source, we tried to suppress *pfkA* in a time-controlled way through exogenous induction and inhibition using CRISPRi system (Fig. 3). The CRISPRi gene regulation system requires only two components, dCas9 protein and a gRNA, to achieve regulation of the transcription level of any gene in the genome. The degree of suppression of gene expression can be controlled by adjusting the binding position and expression amount of the gRNA. Thus CRISPRi is widely used in the field of metabolic engineering and had shown a relatively good inhibition effect [26, 27].

**Fig. 4:**
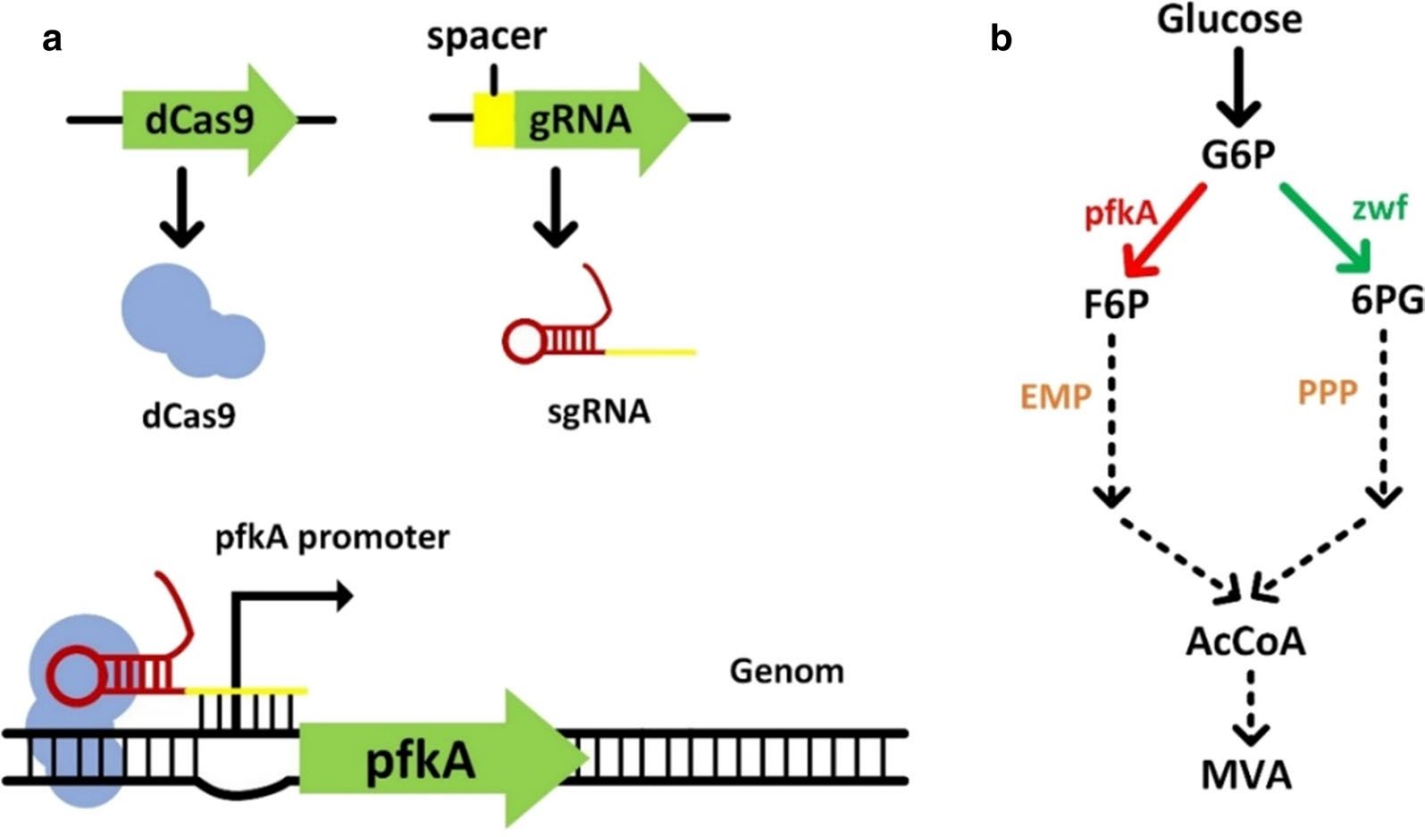
Fermentation of CRISPRi-regulated EP-bifido strains. **a** Glucose consumption of CRISPRi-regulated EP-bifido strains. **b** OD_600_ of CRISPRi-regulated EP-bifido strains. **c** MVA accumulation of CRISPRi-regulated EP-bifido strains. **d** MVA titre and yield of CRISPRi-regulated EP-bifido strains

To avoid the growth inhibition that may be caused by dCas9 from the CRISPRi gene regulation system, we selected a relatively low strength promoter, BBa-J23134, to promote *dcas9*. In order to obtain a different repression range, three different sgRNAs targeting the promoter or coding region of *pfkA* were designed. sgRNA1 were designed on the promoter region of *pfkA*, sgRNA2 and sgRNA3 targeted the coding chain of *pfkA*, at the region of 100 bp and 200 bp downstream of the initial codon, which may cause different repression effect [28]. After *dcas9* and the sgRNAs were incorporated into pFF and pBSA respectively, six CRISPRi-regulated strains were generated. The fermentation results showed that the introduction of CRISPRi significantly inhibited the growth of cells and the glucose consumption was also reduced compared with that of the control strain BW25113 zwf-23100 pFF pBSA (abbreviated to BBF). This may be caused by the toxicity or leaky expression of *dCas9*. The CRISPRi-regulated strains were induced at 12 h by adding IPTG. The fermentation results showed that the three inhibitory sites we selected had different inhibitory effects (Fig. 4). sgRNA1 showed a better inhibition effect. Although strain BW25113 pFF-dCas9 pBSA-sgRNA1 produced only 8.53 g/L MVA, its MVA conversion rate reached 68.7%, which exceeded the previous best MVA conversion rate. Four control strains were also constructed to confirm the effect of the CRISPRi system on cell growth. Strains BW25113 zwf-23100 pBSA-sgRNA1 pFF, BW25113 zwf-23100 pBSA pFF-dCas9, BW25113 zwf-23100 pBSA-sgRNA1-dCas9 pFF and BW25113 zwf-23100 pBSA pFF, were abbreviated to BB1F, BBFd, BB1dF, and BBF, respectively. The results proved that CRISPRi can restrain cell growth effectively in these engineered strains (Additional file 1: Figure S1). The inhibitory effect of sgRNA1 on_*pfkA* is suitable for enhancing MVA fermentation in the EP-bifido system (Fig. 4). In the CRISPRi-regulated strains, we hardly detected any acetic acid, ethanol, or other byproducts during the fermentation process, which was in line with our expectations. The timely inhibition of *pfkA* reduces the flux of glycolysis, so there was no excessive carbon source overflow.

## Discussion

Global warming is mainly due to excess CO_2_ emission; it is urgently necessary to find sustainable solutions to address this issue. Moreover, this wasted carbon may have a major impact on the overall economy of biobased products derived from fermentable carbon sources. Scientists have explored the possibility of using microbial systems to optimize carbon conservation during metabolic processes. The pyruvate decarboxylation step of glycolysis releases CO_2_ into the environment, resulting in 33% loss of carbon yield; this carbon loss has now been challenged by many scientists. We previously constructed an EP-bifido pathway in *E. coli* to reduce CO_2_ emissions and successfully applied it to produce a series of acetyl-CoA-derived compounds such as PHB, MVA and fatty acids. However, in the EP-bifido pathway, metabolic flux distribution between EMP and PPP has great optimization potential for maximum generation of NADPH; in addition, the *pfkA* knockout severely blocked EMP pathway and limited the growth of the engineered strains.

**Fig. 5:**
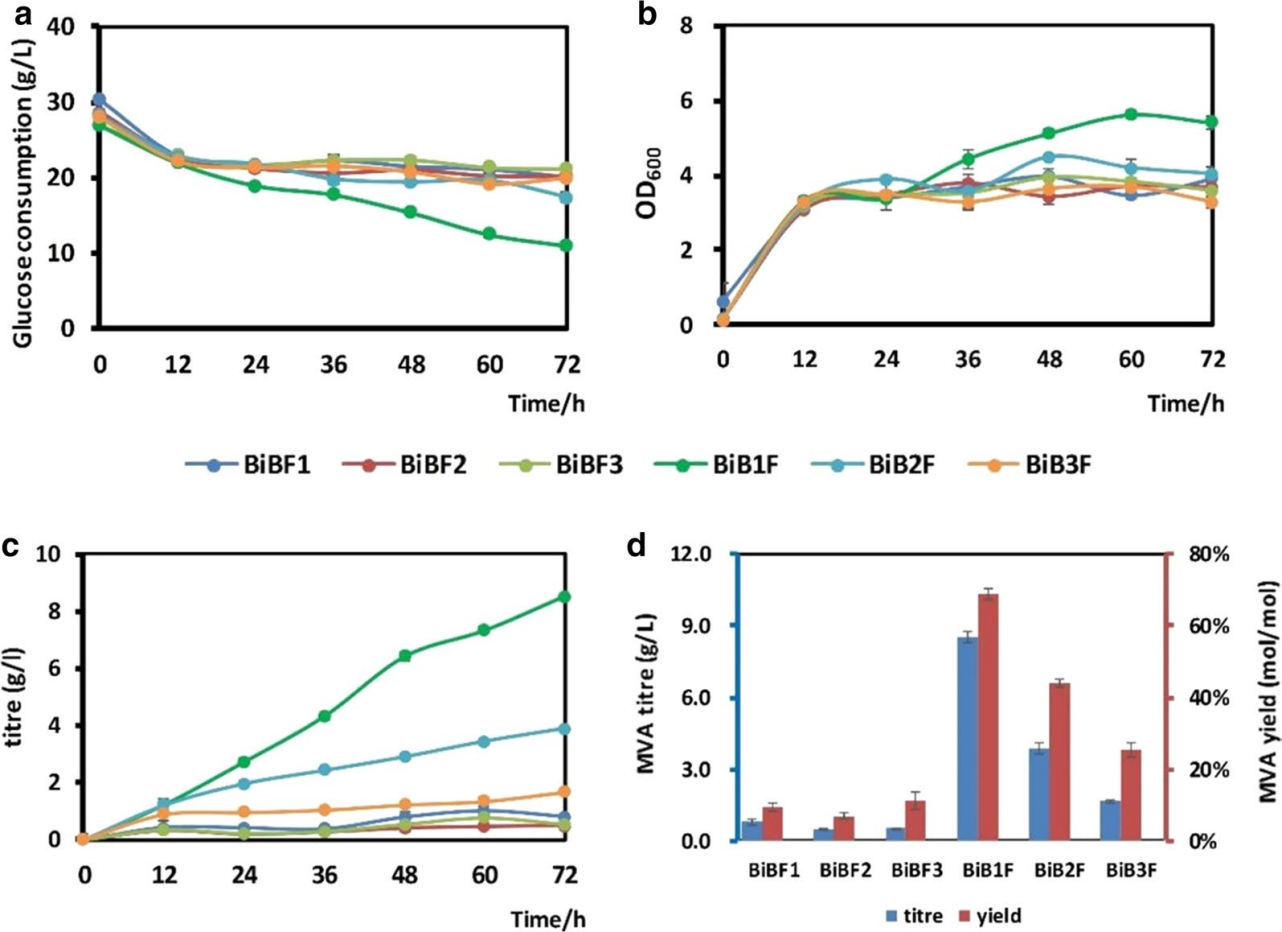
Metabolic flux diagram of *zwf*-strengthened EP-bifido strains. The metabolic flux shown for strains from top to bottom are BW-P10 BF, BW-P08 BF, BW-P BF

Here, our fine-tuning strategy to improve NADPH availability was to enhance the expression of *zwf* by replacing its promoter; we used five promoters with different strengths. The engineered strains BW-P08 BF and BW-P10 BF produced higher MVA titers than the control strain BW-P BF, 9.12 and 11.2 g/L, respectively. The MVA production by these strains did not show an obvious positive correlation with the *zwf* promoter strength*.* Since the MVA yield did not represent the flux distribution between the EMP pathway and the PPP, ^13^C-MFA was performed to detect the metabolic flux distribution in strains BW-P08 BF and BW-P10 BF (which had high MVA titers) and the control strain BW-P BF. The flux ratio between the PPP and the EMP pathway in strains BW-P08 BF and BW-P10 BF was much higher than that in strain BW-P BF (Fig. 5), indicating an improved shunt to the PPP. Also, enhanced *zwf* expression increased the total amount and molar yield of NADPH (Fig. 2). The NADPH-generating pathway shifted from isocitrate dehydrogenase in the TCA cycle to glucose-6-phosphate dehydrogenase in the PPP, further proving that carbon flux was redirected from the EMP pathway to the PPP. The NADH level also showed a decreased TCA cycle activity. In terms of the ATP level, enhancement of *zwf* expression increased the ratio of substrate level phosphorylation (Fig. 2) and reduced the energy supply ratio of pyruvate kinase. Thus, we identified the metabolic flux distribution following the fine-tuning of central metabolic nodes, which helps us to understand the impact on metabolism.

In dynamic regulation of metabolic pathways, the CRISPRi system has recently been used to improve flux through different pathways [29, 30]. One benefit of using the CRISPRi system over promoter replacement methods is that it does not require genome editing of the target gene, which remains a challenge. The introduction of the CRISPRi system is achieved by adding an inducer at a certain time to start the CRISPRi system. Here, we used it to adjust the inhibition level of *pfkA*, so as to achieve timely adjustment of EMP pathway/PPP flux. To reduce the growth inhibition caused by *pfkA* knockout, considering that the strain itself already harbors two plasmids and the CRISPRi system, we integrated *dcas*9 and the sgRNA onto the two plasmids respectively. After introduction of the CRISPRi system, cell growth and sugar consumption of the engineered strains were significantly decreased. The introduction of dCas9 may also have an inhibitory effect on bacterial growth. The relevant limitation of the CRISPRi system is therefore the toxicity of dCas9 expression in certain hosts [31, 32] that would affect the growth of pathway-expressing cells that typically already suffer from growth defects. The three targeting sites played a role in fine-tuning of *pfkA* and sgRNA1 showed the best inhibition effect. The MVA yield of strain BW25113 pFF-dCas9 pBSA-sgRNA1 was only 8.53 g/L, its yield reached 68.7%, exceeding the previous best conversion rate. sgRNA2 and sgRNA3 targeted the coding chain of *pfkA* at the region of 100 bp and 200 bp downstream of the initial codon, while sgRNA1 were designed on the 100 bp upstream of the promoter region of *pfkA*. The design of sgRNA1 can block the binding of RNA polymerase due to the steric hindrance of dcas9; the design of sgRNA2 and sgRNA3 can prevent the elongation of RNA polymerase. The actual inhibitory effects of three sgRNAs on the transcription of *pfkA* were not revealed, however, the strategy of CRISPRi modulation can achieve a broadly gene regulating range through designing various CRISPRi target sites.

The more relevant limitation of the CRISPRi system is the toxicity of dCas9 expression that would affect cell growth. Except for the CRISPRi system, gene repression at the transcriptional level usually combines multiple known promoter systems to construct the inverter to down-regulate genes. Moreover, at post-transcriptional and post-translational level, tools to down-regulate mRNA levels include anti-sense RNA (asRNA) and RNA interference (RNAi). These control methods that function at the post-transcriptional and post-translational levels are useful alternatives. In the future, there must be more convenient methods to down-regulate genes autonomously and dynamically.

## Conclusion

This study showed glycolytic flux ratio fine-tuning strategies applying in an artificial carbon saving pathway for efficient MVA production. The strategies presented in this work serve as a guide to metabolic engineering projects requiring acetyl-CoA as a metabolic precursor.

## Materials and methods

### Media and culture

For plasmid preparation, *E. coli* strains were cultured at 37 °C on a rotary shaker (220 rpm) in test tubes containing 5 mL Luria–Bertani (LB) medium. For MVA production, 50-mL shake flask cultures were started by 2% inoculation from the 5-mL LB culture. The 50-mL cultures contained M9 minimal medium with 0.2% yeast extract containing 20 g/Lglucose and shaken at 37 °C in a rotary shaker (120 rpm) for 48 h. Overnight cultures were shaken at 37 °C in a rotary shaker (220 rpm). Antibiotics were added as follows: ampicillin (Amp) 100 μg/mL, spectinomycin (Spc) 50 μg/mL and chloromycetin (Cm) 25 μg/mL. For promoter integration and replacement procedure, strains were cultivated in SOB medium.

LB medium contains (g/L): tryptone(10), yeast extract(5) and NaCl(10). M9 medium contains (g/L): Na_2_HPO_4_·12H_2_O (15.138), KH_2_PO_4_ (3), NaCl (0.5) and NH_4_Cl (1). SOB medium contains (g/L): tryptone (20), yeast extract (5) and NaCl (5).

### Strains and plasmids

All *E. coli* strains and plasmids used are listed in Table 1. Strain BW-P was used as the starting strain for further genetic manipulation [13]. All primers used for molecular manipulations are listed in Additional file 1: Table S1. All promoter used for genetic manipulation are listed in Table 2.

**Table 1:** Strains and plasmids

Strain and plasmids	Relevant properties	Sources
Strains		
*E. coli* BW25113	F- Δ(*araD-araB*)567, Δ*lacZ4787*(::*rrnB-3*), λ-, rph-1, Δ(*rhaD-rhaB*)568, *hsdR*514	Lab stock
BW-P	BW25113 derivative, Δ*pfkA*::FRT	[13]
BW-P 23100-zwf	BW-P derived, *zwf* promoter:: BBa-J 23100, *CmR*	This study
BW-P 23104-zwf	BW-P derived, *zwf* promoter:: BBa-J 23104, *CmR*	This study
BW-P 23105-zwf	BW-P derived, *zwf* promoter:: BBa-J 23105, *CmR*	This study
BW-P 23108-zwf	BW-P derived, *zwf* promoter:: BBa-J 23108, *CmR*	This study
BW-P 23114-zwf	BW-P derived, *zwf* promoter:: BBa-J 23114, *CmR*	This study
BW-P10 BF	BW-P 23100-zwf carrying pBSA pFF	This study
BW-P04 BF	BW-P 23104-zwf carrying pBSA pFF	This study
BW-P 05 BF	BW-P 23105-zwf carrying pBSA pFF	This study
BW-P 08 BF	BW-P 23108-zwf carrying pBSA pFF	This study
BW-P 14 BF	BW-P 23114-zwf carrying pBSA pFF	This study
BPB10F	BW-P 23100-zwf carrying pBSA-23100 pBSA	This study
BPB02F	BW-P 23100-zwf carrying pBSA-23102 pBSA	This study
BPB04F	BW-P 23100-zwf carrying pBSA-23104 pBSA	This study
BPB18F	BW-P 23100-zwf carrying pBSA-23118 pBSA	This study
BPB19F	BW-P 23100-zwf carrying pBSA-23119 pBSA	This study
BW25113 23100-zwf	BW25113 derived, *zwf* promoter:: BBa-J 23100	This study
BiBF1	BW25113 23100-zwf carrying pBSA-dCas9 pFF-sgRNA1	This study
BiBF2	BW25113 23100-zwf carrying pBSA-dCas9 pFF-sgRNA2	This study
BiBF3	BW25113 23100-zwf carrying pBSA-dCas9 pFF-sgRNA3	This study
BiB1F	BW25113 23100-zwf carrying pBSA-sgRNA1 pFF- dCas9	This study
BiB2F	BW25113 23100-zwf carrying pBSA-sgRNA2 pFF- dCas9	This study
BiB3F	BW25113 23100-zwf carrying pBSA-sgRNA3 pFF- dCas9	This study
BB1F	BW25113 23100-zwf carrying pBSA-sgRNA1 pFF	This study
BBFd	BW25113 23100-zwf carrying pBSA pFF- dCas9	This study
BiBd1F	BW25113 23100-zwf carrying pBSA-sgRNA1-dCas9 pFF	This study
pFF	pCDFduet, with fxpk from *B. Adolescentis*, and *fbp* gene from *E. coli*, Amp^R^	[13]
pBSA	pTrc99a with *atoB* from *E. coli*, *mvaS* and *mvaA* from *L. casei.* tac promoter, Amp^R^	[13]
pTKRED	ParaBAD promoter containing plasmid, Spe^R^	[34]
pCP20	Helper plasmid expressing FLP recombinase, ts-rep, Amp^R^, Cm^R^	[35]
pKD3	Template plasmid with Cm^R^ gene and FLP recognition target	[35]
P23100-GFP	pTrc99a with BBa-J 23100 promoter and green fluorescent protein sequence, Amp^R^	This study
P23104-GFP	pTrc99a with BBa-J 23104 promoter and green fluorescent protein sequence, Amp^R^	This study
P23105-GFP	pTrc99a with BBa-J 23105 promoter and green fluorescent protein sequence, Amp^R^	This study
P23108-GFP	pTrc99a with BBa-J 23108 promoter and green fluorescent protein sequence, Amp^R^	This study
P23114-GFP	pTrc99a with BBa-J 23114 promoter and green fluorescent protein sequence, Amp^R^	This study
Pzwf-GFP	pTrc99a with *zwf* promoter from *E. coli* BW25113 and green fluorescent protein sequence, Amp^R^	This study
pBSA-23119	pBSA with BBa-J 23119 promoter	This study
PBSA-23100	pBSA with BBa-J 23100 promoter	This study
PBSA-23102	pBSA with BBa-J 23102 promoter	This study
PBSA-23104	pBSA with BBa-J 23104 promoter	This study
PBSA-23118	pBSA with BBa-J 23118 promoter	This study
pFF-sgRNA1	pFF containing *pfkA* sgRNA1, Spc^R^	This study
pFF-sgRNA2	pFF containing *pfkA* sgRNA2, Spc^R^	This study
pFF-sgRNA3	pFF containing *pfkA* sgRNA3, Spc^R^	This study
pBSA-dCas9	pBSA containing Cas9 array, Amp^R^	This study
pBSA-sgRNA1	pBSA containing *pfkA* sgRNA1, Amp^R^	This study
pBSA-sgRNA2	pBSA containing *pfkA* sgRNA2, Amp^R^	This study
pBSA-sgRNA3	pBSA containing *pfkA* sgRNA3, Amp^R^	This study
pFF-dCas9	pFF containing Cas9 array, Amp^R^	This study
pFF-sgRNA1-dCas9	pFF containing Cas9 array and *pfkA* sgRNA1, Amp^R^	This study
pBSA-23102-sgRNA1	pBSA-23102 containing *pfkA* sgRNA1, Amp^R^	This study

**Table 2:** Relative strength of Promoters

Promoter	Relative strength
BBa-J23119	–
BBa-J23100	1.0
BBa-J23102	0.86
BBa-J23104	0.72
BBa-J23105	0.51
BBa-J23108	0.24
BBa-J23134	0.18
BBa-J23114	0.1

‘–’ The promoter strength of BBa-J23119 was not detected

### Plasmid construction for MVA production

To replace the original tac promoter of pBSA plasmid, five promoters BBa-J 23119, BBa-J 23100, BBa-J 23102, BBa-J 23104, BBa-J 23118 was designed into primers to construct plasmids pBSA-23119, pBSA-23100, pBSA-23102, pBSA-23104, pBSA-23118. Two primers were designed in the opposite direction. Five PCR amplicons were obtained using the original plasmid as the template with primier pcr-23119-F/R, pcr-23100-F/R, pcr-23102-F/R, pcr-23104-F/R, pcr-23118-F/R. *Dpn*I was added to the PCR system in 37 °C for 1 h to remove the methylated template. A mixture of 50 μL was transformed into competent cells using chemical transformation. Colony PCR was performed by picking monoclonal from resistance plate to eliminate false positives and template interference. Finally, the five plasmids were transformed into the BW-P 23100 strain with the plasmid pFF.

### Construction of CRISPRi suppression system

To select the CRISPRi inhibition site, three different sgRNAs was designed by targeting *pfkA* promoter sequence, 100 bp downstream of the promoter sequence, and 200 bp downstream of the initiation codon. *dcas9* and sgRNAs were assembled into pFF and pBSA plasmids respectively downstream of an IPTG-induced promoter. Primer dCas9FF-F/R, dCas9-F/R were used to amplify dCas9 sequence. Primer sgRNA-F1, sgRNA1-R and sgRNA-F2, sgRNA1-R were used to amplify sgRNA1 sequence by PCR. The two amplified sequences were overlapped from homology arms. Sequences sgRNA2 and sgRNA3 were amplified as above. All constructed plasmids were electro-transformed into *E. coli* strains. To accomplished the timely control of *pfkA* by CRISPRi system, cells was induced by 200 μM IPTG after 12 h of fermentation.

### Promoter replacement on *E. coli* genome

Promoter for replacing the promoter of *zwf* gene were selected from the Anderson promoter library (http://parts.igem.org/Promoters/Catalog/Anderson). Promoter replacement primers homoarm-F and homoarm-cm-R were designed using homology arms at about 300–500 bp at both ends of the target gene promoter, and plasmid pKD3 or pKD4 was used as a template to obtain recombinant fragments with kan or Cm resistance by using PKD3-cm-F/R or PKD4-cm-F/R. Three amplified sequences were overlapped from homology arms and resistance tag. All five replacing sequences were amplified as above.

The Red homologous recombination method was employed for gene integration. The pTKRED complementary plasmid was transformed into the target strain. The electrotransfection were performed by growing BW-P in 5 mL LB medium at 30 °C and shaking at 220 rpm for 12 h. 5-mL shake flask cultures using SOB broth were started with a 1% inoculation from the overnight culture. Isopropyl-β-D-thiogalactopyranoside (IPTG) was added to a final concentration of 0.5 mM to induce λ-prophage (bet, gam, and exo) gene expression. Cells were then incubated at 30 °C and shaking at 220 rpm until reaching an OD_600_ of 0.5 to 0.6. Cell were collected (2 mL), pelleted, and washed three times with cold sterile water to make them electrocompetent. ssDNA mixture (1 μM) was added to electrocompetent cells and electroporated at 2.5 kV. Add 1 mL LB liquid medium and cultivate for 1 h. After centrifuged, the collected bacteria were plated onto plates containing 25ug/ml kan or 18ug/ml spc for overnight incubation at 37 °C. Transformed strains were selected by their kanR phenotype and were verified by PCR.

### Measurement of extracellular metabolites

A spectrophotometer was used to measure the optical density at 600 nm (OD_600_) of the bacterial culture. For extracellular metabolite analysis, 1 mL of culture was centrifuged at 12,000*g* for 2 min. The supernatant was filtered through a 0.22-μm syringe filter for high-performance liquid chromatography analysis. Glucose, MVA, acetate, and pyruvate were measured on an ion exchange column (HPX-87H; Bio-Rad Labs) with a differential refractive index detector (Shimadzu RID-10A). A 0.5-mL/min mobile phase using a 5 mM H_2_SO_4_ solution was applied to the column. The column was operated at 65 °C.

### ^13^C-MFA

To investigate if the carbon flux was really redistributed to the newly constructed EP-bifido pathway, 13C-MFA was performed using 100% 1-^13^C_1_ glucose as the feeding substrate was added to a concentration of 10 g/L. Cells at the exponential growth phase were harvested by centrifugation at 7000 g for 5 min at 4 °C. The cell pellet was then washed twice with chemical defined medium and hydrolyzed in 6 M HCl for 24 h at 120 °C (Schwender et al. 2006). The resulting proteinogenic acids were derivatized with N-(tert-butyldimethylsilyl)-N-methyl-trifluoroacetamide containing tert-butyldimethylchlorosilane in acetonitrile at 105 °C for 1 h, and then analyzed by a GC–MS [Agilent 7890 A GC and 5975 C Mass Selective Detector (Agilent Technologies, Santa Clara, USA)] equipped with a DB-1column (Agilent Technologies). The data obtained from GC–MS were corrected by reduction of the natural abundance ratio of C, H, O, N, and Si isotopes [30]. Metabolic fluxes were estimated by minimizing the residual sum of squares between experimentally measured and model predicted ^13^C-enrichment using ^13^C-Flux software obtained from Dr. Wiechert [33].

## Supplementary Information

**Additional file 1: Figure S1.** Fermentation of a series of CRISPRi-control strains. ** Table S1** Primers used in this study.

**Additional file 2.** MFA simulated result.
