# Supplementary material for: Fine tuning the glycolytic flux ratio of EP-bifido pathway for mevalonate production by enhancing glucose-6-phosphate dehydrogenase (Zwf) and CRISPRi suppressing 6-phosphofructose kinase (PfkA) in Escherichia coli
Source: Microb Cell Fact. 2021 Feb 2;20:32. doi: 10.1186/s12934-021-01526-1 (PMC7852082; doi:10.1186/s12934-021-01526-1)
Supplement: Supplementary file 1 — Additional file 1: Figure S1. Fermentation of a series of CRISPRi-control strains. Table S1 Primers used in this study. [file 12934_2021_1526_MOESM1_ESM.docx]

Fine and dynamic tuning the glycolytic flux ratio of an artificial carbon saving pathway for high yield of mevalonate in *Escherichia coli*

Ying Li^1†^, He Xian^3†^, Ya Xu^1^, Yuan Zhu^1^, Zhijie Sun^2*^, Qian Wang^1*^, Qingsheng Qi^1^.

^1^National Glycoengineering Research Center, State Key Laboratory of Microbial Technology, Shandong University, Qingdao, PR China

^2^Marine Biology Institute, Shantou University, Shantou, PR China

^3^School of Chemistry and Molecular Biosciences, Faculty of Science, The University of Queensland, Australia.

**Additional file 1: figures and tables**


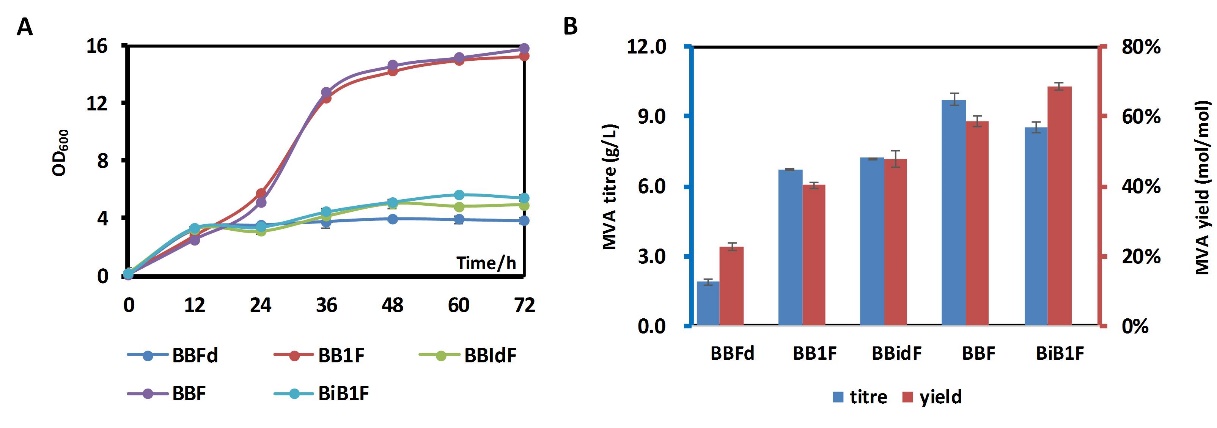


**Figure S1 Fermentation of a series of CRISPRi-control strains.**

A: OD_600_ of CRISPRi-control strains. B: MVA titre and yield of CRISPRi-control strains

BB1F：BW25113 zwf-23100 pBSA pFF-dCas9,

BBFd：BW25113 zwf-23100 pBSA pFF-dCas9,

BB1dF：BW25113 zwf-23100 pBSA-sgRNA1-dCas9 pFF,

BBF：BW25113 zwf-23100 pBSA pFF,

BiB1F：BW25113 zwf-23100 pBSA-sgRNA1 pFF-dCas9.

**Table S1 Primers used in this study.**

| **Primer name** | **Sequence** |  |
| --- | --- | --- |
| homoarm-F | 5'-CGCGGTTGATGGCAGATTTAT-3' | |
| homoarm-cm-R | 5'-AGACGTGTAATGCTGCAATCTCTGACACTGATCATGTTATG-3' | |
| cm-23110-F | 5'-CATAACATGATCAGTGTCAGAGATTGCAGCATTACACGTCT-3' | |
| 23110-cm-R | 5'-GCGCGCAAAGTGCGGCAAAACTGATAAAAAAATCATCGTTTTCCATTGGGTAAAAACATGGGAATTAGCCATGGTCC-3' | |
| 23110-zwf-F1 | 5'-CACGGGTGGATAAGCGTTTGACGGCTAGCTCAGTCCTAGGTACAGTGCTAGCGTAAGAAAATTACAAGTATACCCTGGC-3' | |
| 23110-zwf-F2 | 5'-CGATGATTTTTTTATCAGTTTTGCCGCACTTTGCGCGCTTTTCCCGTAATCGCACGGGTGGATAAGCGTTTGAC-3' | |
| zwf-R | 5'-GTTATTCACAAACAGGGAGTTAGC-3' | |
| TESTp-F1 | 5'-AGAACTTATTCATCGCATCGTGG-3' | |
| TESTp-R1 | 5'-CTCCTTAAGTTAACTAACCCGGTAC-3' | |
| TESTp-F2 | 5'-CCGGTATCAACAGGGACACC-3' | |
| TESTp-R2 | 5'-ATCAATGTCGACGCGGATCGC-3' | |
| 23104-zwf-F1 | 5'-CACGGGTGGATAAGCGTTTGACAGCTAGCTCAGTCCTAGGTATTGTGCTAGCGTAAGAAAATTACAAGTATACCCTGGC-3' | |
| 23105-zwf-F1 | 5'-CACGGGTGGATAAGCGTTTTACGGCTAGCTCAGTCCTAGGTACTATGCTAGCGTAAGAAAATTACAAGTATACCCTGGC-3' | |
| 23105-zwf-F2 | 5'-CGATGATTTTTTTATCAGTTTTGCCGCACTTTGCGCGCTTTTCCCGTAATCGCACGGGTGGATAAGCGTTTTAC-3' | |
| 23108-zwf-F1 | 5'-CACGGGTGGATAAGCGTCTGACAGCTAGCTCAGTCCTAGGTATAATGCTAGCGTAAGAAAATTACAAGTATACCCTGGC-3' | |
| 23108-zwf-F2 | 5'-CGATGATTTTTTTATCAGTTTTGCCGCACTTTGCGCGCTTTTCCCGTAATCGCACGGGTGGATAAGCGTCTGAC-3' | |
| 23114-zwf-F1 | 5'-CACGGGTGGATAAGCGTTTTATGGCTAGCTCAGTCCTAGGTACAATGCTAGCGTAAGAAAATTACAAGTATACCCTGGC-3' | |
| 23114-zwf-F2 | 5'-CGATGATTTTTTTATCAGTTTTGCCGCACTTTGCGCGCTTTTCCCGTAATCGCACGGGTGGATAAGCGTTTTAT-3' | |
| CUT-sGFP-F | 5'-ACCATGGAATTCGAGCTCGGTACCCGGTTACGCCGCCAGCGCATAG-3' | |
| 23110-sGFP-R | 5'-TGAAAATCTTCTCTCATCCGCCAAAACAGCCTTGACGGCTAGCTCAGTCCTAGG-3' | |
| 23104-sGFP-R | 5'-TGAAAATCTTCTCTCATCCGCCAAAACAGCCTTGACAGCTAGCTCAGTCCTAGG-3' | |
| 23105-sGFP-R | 5'-TGAAAATCTTCTCTCATCCGCCAAAACAGCCTTTACGGCTAGCTCAGTCCTAGG-3' | |
| 23108-sGFP-R | 5'-TGAAAATCTTCTCTCATCCGCCAAAACAGCCCTGACAGCTAGCTCAGTCCTAGG-3' | |
| 23114-sGFP-R | 5'-TGAAAATCTTCTCTCATCCGCCAAAACAGCCTTTATGGCTAGCTCAGTCCTAGG-3' | |
| zwf O -sGFP-R2 | 5'-TTCGCAAGCTCGTAAAAGCAGTACAGTGCACCACTAGTGATTAAAGAGGAGAAATACTAG -3' | |
| PCR-vector-F | 5'-GGCTGTTTTGGCGGATGAGAGAAGATTTTCA-3' | |
| PCR-vector-R | 5'-CCGGGTACCGAGCTCGAATTCCATGGT-3' | |
| GFP-test-F | 5'-TCATCGACTGCACGGTGCAC-3' | |
| GFP-test-R | 5'-TGCCTGGCAGTTCCCTACTCT-3' | |
| PKD3-cm-F | 5'-TTGAGCGATTGTGTAGGCTGGAGCTGCTTC-3' | |
| PKD3-cm-R | 5'-CATATGAATATCCTCCTTAGTTCCTATTCC-3' | |
| pcr-23118-F | 5'-TTGACGGCTAGCTCAGTCCTAGGTATTGTGCTAGCTGTGGAATTGTGAGCGGATAACAATTTCAC-3' | |
| pcr-23118-R | 5'-GCTAGCACAATACCTAGGACTGAGCTAGCCGTCAACAGCTCATTTCAGAATATTTGCCAGAACC-3' | |
| pcr-23102-F | 5'-TTGACAGCTAGCTCAGTCCTAGGTACTGTGCTAGCTGTGGAATTGTGAGCGGATAACAATTTCAC-3' | |
| pcr-23102-R | 5'-GCTAGCACAGTACCTAGGACTGAGCTAGCTGTCAACAGCTCATTTCAGAATATTTGCCAGAACC-3' | |
| pcr-23104-F | 5'-TTGACAGCTAGCTCAGTCCTAGGTATTGTGCTAGCTGTGGAATTGTGAGCGGATAACAATTTCAC-3' | |
| pcr-23104-R | 5'-GCTAGCACAATACCTAGGACTGAGCTAGCTGTCAACAGCTCATTTCAGAATATTTGCCAGAACC-3' | |
| pcr-23100-F | 5'-TTGACGGCTAGCTCAGTCCTAGGTACAGTGCTAGCTGTGGAATTGTGAGCGGATAACAATTTCAC-3' | |
| pcr-23100-R | 5'-GCTAGCACTGTACCTAGGACTGAGCTAGCCGTCAACAGCTCATTTCAGAATATTTGCCAGAACCG-3' | |
| pcr-23119-F | 5'-TTGACAGCTAGCTCAGTCCTAGGTATAATGCTAGCTGTGGAATTGTGAGCGGATAACAATTTC-3' | |
| pcr-23119-R | 5'-GCTAGCATTATACCTAGGACTGAGCTAGCTGTCAACAGCTCATTTCAGAATATTTGCCAGAACC-3' | |
| dCas9FF-F | 5'-CCGTAGCGCCGATGGTAGTGTGGGGTCTCCCCATGCGAGAGTAGGG-3' | |
| dCas9FF-R | 5'-TTATCCGCTCACAATTCCACAGCTAGCATTGTACCAAGGGCTGAGCTAGCTATAAACGTTTCACTTCTGAGTTCGGCAT-3' | |
| dCas9-F | 5'-AGCTGTGGAATTGTGAGCGGATAACAATTTCACACAGGAAACAGACATGGATAAGAAATACTCAATAGGCTTAGCTATC-3' | |
| dCas9-R | 5'-CCCCACACTACCATCGGCGCTACGGTCAGTCACCTCCTAGCTGACTC-3' | |
| sgRNA-F1 | 5'-AGGCTAGTCCGTTATCAACTTGAAAAAGTGGCACCGAGTCGGTGCTTTTTTTGGAGCAGGACGCCCGCCATAAAC-3' | |
| sgRNA-F2 | 5'-GTTTTAGAGCTAGAAATAGCAAGTTAAAATAAGGCTAGTCCGTTATCAACTTG-3' | |
| sgRNA-R | 5'-CCGTTGGCTAGCATTATACCTAGGACTGAGCTAGCTGTCAGCTTCGCAACGTTCAAATCCG-3' | |
| sgRNA3-R | 5'-CTAGCCTTATTTTAACTTGCTATTTCTAGCTCTAAAACTATGGTACAGCTAGACCGTTCCGTTGGCTAGCATTATACCT-3' | |
| sgRNA2-R | 5'-CTAGCCTTATTTTAACTTGCTATTTCTAGCTCTAAAACCAATTCGCGGGGTTGTTCGTCCGTTGGCTAGCATTATACCT-3' | |
| sgRNA1-R | 5'-CTAGCCTTATTTTAACTTGCTATTTCTAGCTCTAAAACAAGTTCAGAGGTAGTCATGACCGTTGGCTAGCATTATACCT-3' | |
| BSA-sgRNA-test-F | 5'-GAAGTGAAACGCCGTAGCGC-3' | |
| FF-dCas9-test-F | 5'-CTCTACCCAAGCACCGCCAG-3' | |
| test-sgRNA-R | 5'-GTCGGCAACGACCATATGGTTG-3' | |
| test-sgRNA-F | 5'-GCAGTGTGACCGTGTGCTTC-3' | |
